# Leukocytes Transcriptome Analysis of Genes Associated with Epilepsy Duration and Age of Onset

**DOI:** 10.1007/s12035-026-05845-5

**Published:** 2026-04-08

**Authors:** Mayuresh Anant Sarangdhar, Johan Zelano

**Affiliations:** 1 Department of Clinical Neuroscience, Institute of Neuroscience and Physiology, Sahlgrenska Academy, University of Gothenburg, Blå Stråket 7, Plan 3, Sahlgrenska Sjukhuset, 41345 Gothenburg, Sweden; 2 Wallenberg Center of Molecular and Translational Medicine, University of Gothenburg, Gothenburg, Sweden; 3 Department of Neurology, Sahlgrenska University Hospital, Member of ERN Epicare, Gothenburg, Sweden

**Keywords:** Epilepsy duration, Epilepsy onset-age, Biomarker, Leukocytes

## Abstract

Epilepsy is increasingly understood not as a fixed condition, but a dynamic and potentially progressive disorder. Beyond its primary impact on the brain, growing evidence indicates that epilepsy also exerts widespread systemic effects, influencing multiple physiological systems. Two temporal factors—the age at seizure onset and duration of epilepsy—could help understand if time with epilepsy shapes systemic molecular changes and in turn provide new insights into disease progression and therapeutic resistance. To identify systemic signatures associated with age of onset and epilepsy duration, we investigated the molecular correlates of them using leukocyte transcriptome. Differential expression analyses of short (≤ 20 years) vs. long (> 20 years) disease duration and childhood (≤ 12 years) vs. adolescent (> 12 years) onset-age revealed distinct leukocyte transcriptomics signatures. Gene expression changes related to disease duration were enriched for pathways involved in oxidative stress and disruptions in RNA/DNA regulatory processes. Epilepsy onset was linked to enrichment of protein demannosylation, deglycosylation and immune-related pathways, particularly those involving the MHC protein complex and antigen presentation. Spearman’s correlation analyses uncovered genes whose expression correlated with age of onset and duration. Additionally, we found many differentially expressed genes located within the EJM1 locus on chromosome 6p21, the known major susceptibility locus for juvenile myoclonic epilepsy (JME). This finding suggests that co-regulated gene networks within this locus may act synergistically to influence neuroimmune interactions relevant to both the development and progression of epilepsy. The study suggests dynamic immune-related transcriptomic changes in epilepsy, which may warrant further investigation as potential biomarkers of disease onset and progression.

**Supplementary Information:**

The online version contains supplementary material available at 10.1007/s12035-026-05845-5.

## Introduction

Epilepsy affects over 50 million people worldwide. It remains a heterogeneous disorder with variable clinical trajectories. Age at seizure onset and duration of epilepsy are two routinely recorded but underutilized clinical parameters that may hold key insights into the biological underpinnings and clinical trajectory of the disease. These two factors influence treatment response, cognitive outcomes, and surgical candidacy. Despite accumulating evidence that epilepsy is a dynamic and potentially progressive condition, the molecular correlates of childhood vs. adolescent onset and short vs. long disease duration remain poorly understood. Neuroimaging studies have begun to illuminate the progressive nature of epilepsy, revealing that longer disease duration is associated with widespread reductions in glucose metabolism, particularly in temporal lobe epilepsy (TLE). This hypometabolism, initially confined to the epileptogenic temporal cortex, appears to extend to adjacent cortical regions over time, suggesting a gradual spread of dysfunction [1, 2]. Region-specific effects have also been observed—early seizure onset correlates with hippocampal hypometabolism, while longer duration is linked to amygdalar involvement [3]. Collectively, these findings highlight a progressive decline in brain glucose metabolism associated with chronic epilepsy.

At the molecular level, prolonged epilepsy duration has been linked with dynamic neurobiological adaptations in the brain. For example, hippocampal 5-HT1A receptor (5-HT1AR) density has been shown to correlate with epilepsy duration in patients with TLE with hippocampal sclerosis [4]. In another study, DNA methylation profiling of hippocampal and neocortical tissues revealed widespread and duration-dependent changes, particularly within inflammation-related pathways, implicating sustained molecular remodeling over the course of the disease [5].

Emerging evidence suggests that both epilepsy duration and age at seizure onset are associated with progressive systemic alterations beyond the brain. For instance, longer epilepsy duration has been identified as an independent risk factor for electrocardiographic (ECG) abnormalities in both pediatric and adult populations, indicating potential cumulative cardiac effects of chronic epilepsy [6, 7]. Another important systemic function is respiration, which may also be influenced by age at seizure onset. Older age at the onset of epilepsy has been linked to longer periods of peri-ictal oxygen desaturation, suggesting that the timing of seizure initiation can shape peripheral physiological responses to seizures [8, 9]. These findings point to a broader impact of epilepsy duration and onset-age on systemic functions, potentially reflecting underlying molecular dysregulation over time. In addition to physiological changes, prolonged epilepsy duration and childhood onset have also been linked to significant behavioral and cognitive challenges. Behavioral problems are notably more prevalent in individuals with epilepsy duration exceeding five years, with long-standing epilepsy and early onset contributing to difficulties in social integration and academic placement [10]. Cognitive impairment in TLE has similarly been associated with longer disease duration, suggesting that the chronicity of epileptic activity itself may progressively affect neural circuits underlying cognition [11]. In pediatric populations, especially children with tuberous sclerosis complex (TSC), extended epilepsy duration and increased antiepileptic drug burden are strongly associated with delays in language development [12]. Collectively, these findings reinforce the notion that both the timing of seizure onset and the cumulative burden of epilepsy contribute to widespread systemic and neurodevelopmental disruptions over time.

Importantly, epilepsy duration and age at onset have prognostic value. A shorter epilepsy duration has consistently been associated with more favorable seizure outcomes following resective epilepsy surgery, underscoring the potential benefits of early surgical intervention in appropriate candidates [13]. Similarly, in patients undergoing neuromodulatory therapy such as vagus nerve stimulation (VNS), epilepsy duration has been identified as an independent predictor of therapeutic response. Specifically, individuals with a disease duration of less than 12.5 years—particularly those between 5 and 12.5 years—are significantly more likely to respond to VNS [14]. These findings emphasize the value of considering disease chronicity and onset timing not only for prognosis but also for personalizing treatment strategies, highlighting their potential utility as biomarkers in guiding clinical decision-making.

Additionally, childhood onset epilepsy (ChOE), often tied to genetic or developmental causes—can interfere with brain maturation and contribute to persistent cognitive and behavioral deficits [15]. In contrast, adolescent onset epilepsy (AOE), is more frequently associated with environmental triggers, or acquired factors, and may show different patterns of seizure types and cognitive outcomes compared to ChOE. Comparing ChOE vs. AOE and short vs. long disease duration provides a unique opportunity to investigate how epilepsy evolves across the lifespan, and to identify critical windows for intervention.

In this exploratory study, we investigated the blood leukocyte transcriptome to identify molecular patterns associated with epilepsy duration and age at seizure onset. Understanding these molecular changes may help identify new biomarkers for disease monitoring and targets for therapeutic intervention across the epilepsy lifespan.

## Method

### RNA-seq Data Processing

Publicly available leukocyte RNA-seq data from 16 epilepsy patients (GEO accession: “GSE217726”) [16, 17] was obtained via “GEO RNA-seq Experiments Interactive Navigator” (GREIN) platform [18], https://www.ilincs.org/apps/grein/?gse = . Gene-level normalized expression data (CPM and TMM) and sample metadata were downloaded directly from GREIN (file: GSE217726_GeneLevel_Normalized(CPM.and.TMM)_data.csv). Additional clinical metadata collected from original publication associated with the dataset. Using custom python script low expressed genes were filtered retaining genes with CPM > 1 in at least 50% of samples. The filtered expression data was log transformed. To avoid undefined values resulting from log (0), a pseudocount of 0.00002828 (the smallest non-zero expression value in the dataset/1000) was added to all expression values prior to log transformation.

### Differential Expression Analysis

Differential gene expression analysis was conducted to compare groups stratified by epilepsy duration (long: > 20 years vs. short: ≤ 20 years) and age at seizure onset (childhood: ≤ 12 years vs. adolescent: > 12 years). Genes were classified as differentially expressed if p value < 0.05 and |log₂ fold change|> 0.5. Results were visualized using volcano plots, where -log₁₀(p-value) was plotted against log₂ fold change to highlight significantly up- and downregulated genes.

### Functional Association Network Analysis

List of upregulated and downregulated genes were separately provided as an input in https://genemania.org/ to predict the functions of gene sets [19]. Gene symbols not recognized by GeneMANIA were removed. “Maximum resultant genes” and “maximum resultant attributes” set at 10. For gene set where no significant functional associations were identified under these restrictive parameters, the maximum number of resultant genes and attributes was increased to 20. “Automatically selected weighting method” used for network weighting. The top four functions based on FDR are presented in the figure.

### Correlation Analyses

To assess gene associations with clinical variables, Spearman’s rank correlation coefficient (r) was calculated between expression levels of each gene (CPM) and epilepsy duration. Genes with |r|> 0.5 and *p*-values < 0.05 were considered strongly correlated. For age at seizure onset, Spearman correlation was similarly applied. Since age is a major determinant of gene expression and represents an important potential confounder, to address this age-associated genes (1497 age associated genes from whole-blood gene expression meta-analysis [20]) were excluded prior to visualization of correlation results ensuring that plotted associations reflect epilepsy duration/onset age-specific effects.

To adjust for potential confounding clinical factors, partial Spearman correlations were calculated using residuals from linear regression models via custom python script. Specifically, expression values and clinical traits (e.g., duration or onset age) were each regressed against covariates including age at sampling, gender, and epilepsy etiology. The correlation between the residuals represented the covariate-adjusted association. This approach allowed for more accurate identification of genes independently associated with clinical disease course. To visualize partial Spearman correlations, residual-residual plots were used. Scatter plots and regression lines were generated using residualized values, ensuring that the visual trend accurately reflects the direction and magnitude of the partial correlation after covariate adjustment.

### WebGestalt Over-Representation Analysis

Functional over-representation analysis was performed using WebGestalt (https://www.webgestalt.org/) [21]. Over-representation analysis selected as a method of interest. Chromosomal location was selected as the functional database, with enrichment assessed at the level of cytogenetic bands. The human genome was used as the reference set. The input gene list consisted of significantly upregulated and downregulated genes combined, allowing unbiased assessment of chromosomal enrichment associated with disease-related transcriptional changes.

## Results

### Leukocyte Transcriptome Changes with Epilepsy Duration

**Fig. 1:**
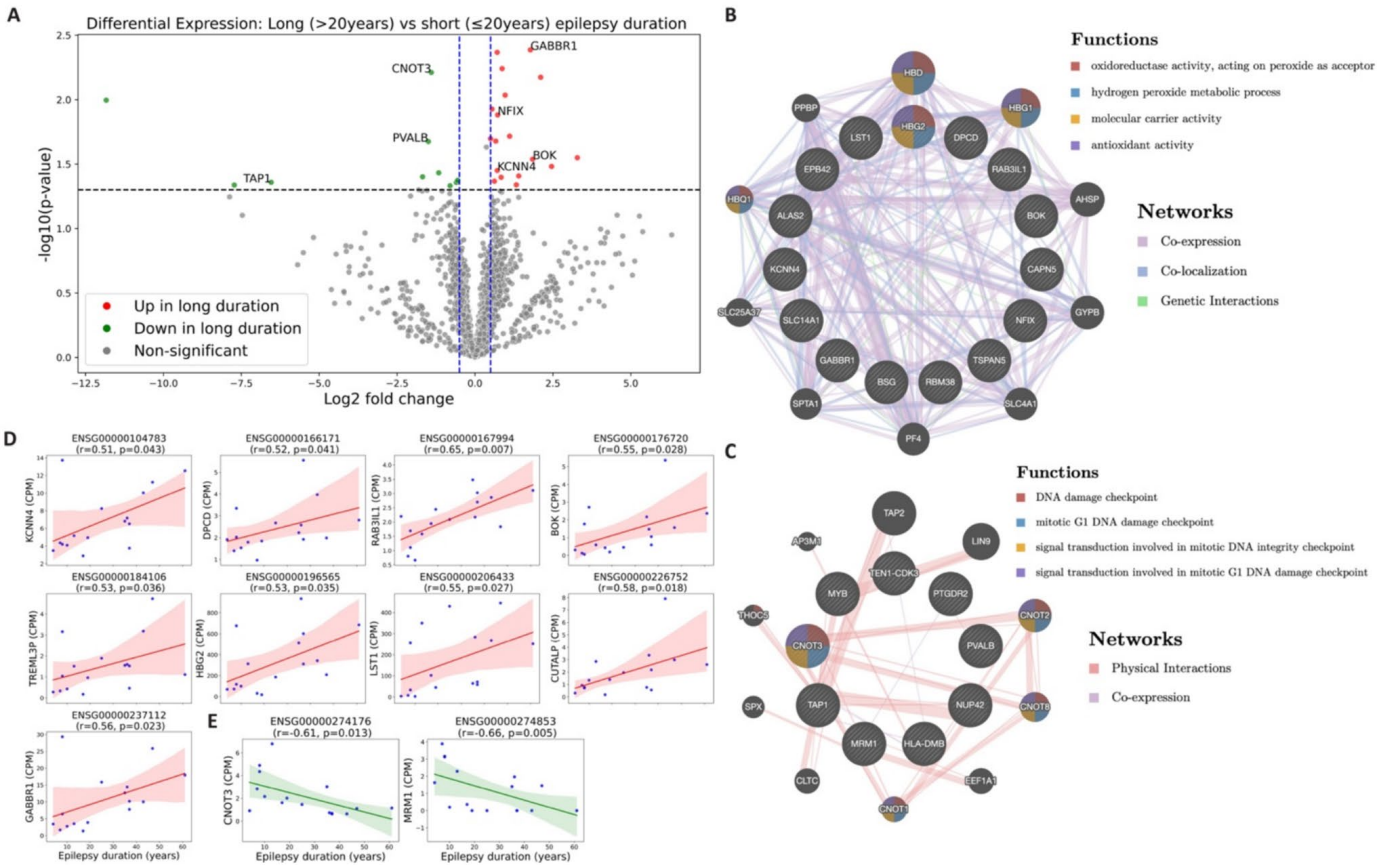
Leukocyte transcriptomic alterations associated with epilepsy duration. (**A**) Volcano plot showing differentially expressed genes in whole-blood leukocytes between patients with short-duration (≤ 20 years) and long-duration (> 20 years) epilepsy. Significantly upregulated genes in the long-duration group (log₂ fold change > 0.5, *p*-value < 0.05) are highlighted in red, while significantly downregulated genes are shown in green. Selected genes previously implicated in epilepsy are annotated. (**B-C**) Functional association network analysis of significantly upregulated (**B**) and downregulated (**C**) genes using GeneMANIA (https://genemania.org/). Input (query) genes are indicated by striped inner nodes. The top four enriched functional terms (based on FDR) are displayed. (**D-E**) Spearman correlations between expression levels of upregulated (**D**) and downregulated genes (**E**) and disease duration. Only non-age-associated genes were visualized. Red and green lines indicate linear regression, and shaded areas represent 95% confidence intervals. *r*: Spearman correlation coefficient

Leukocyte transcriptomes from 16 temporal lobe epilepsy patients (mean age 39.4 years, range 16–62 years; 10 males and 6 females) were analyzed to investigate transcriptomic changes associated with epilepsy chronicity. On average, approximately 11600 genes were detected per individual. After applying expression filtering (CPM ≥ 1 in at least 50% of samples), 11929 genes were retained for downstream analyses. Patients were stratified into two groups based on epilepsy duration: short-duration: ≤ 20 years and long-duration: > 20 years, with 8 patients in each group. The mean epilepsy duration was 10.75 years in the short-duration group and 40.12 years in the long-duration group. As expected, age differed significantly between the groups, while baseline seizure frequency was comparable (Table 1 and 2). Age at seizure onset did not reach statistical significance but showed a strong trend toward earlier onset in the long-duration group compared with the short-duration group (mean onset age: 9.13 vs. 18.88 years; *p* = 0.0515) (Tables 1 and 2). Differential gene expression analysis of whole-blood leukocyte RNA-seq data identified 18 genes significantly upregulated (log₂ fold change > 0.5) and 10 genes significantly downregulated in the long-duration group compared to the short-duration group (Fig. 1A, Suppl. data file A and B). Some of these differentially expressed genes have previously been implicated in epilepsy for example, GABBR1, CNOT3 and KCNN4. Functional association network analysis of upregulated genes revealed involvement in oxidoreductase activity, hydrogen peroxide metabolic process and antioxidant activity (Fig. 1B), indicating enhanced oxidative stress signaling in chronic epilepsy. In contrast, downregulated genes were enriched in DNA damage checkpoint pathways and mRNA catabolic processes (Fig. 1C), which may indicate potential impairments in transcriptomic fidelity and genomic stability with prolonged disease duration. Interestingly, we identified four genes—LST1, TAP1, HLA-DMB and GABBR1—that were significantly differentially regulated with epilepsy duration, all of which are located within the EJM1 locus on chromosome 6p21, a genomic region previously established as a major susceptibility locus for juvenile myoclonic epilepsy (JME) [22] (supp. Figure 1A). The differential regulation of these genes in chronic epilepsy suggests importance of EJM1 region not only in epilepsy susceptibility but also in its temporal dynamics, although further studies are needed to clarify this relationship.

**Table 1:** Clinical metadata of patients

Sample GEO accession	Baseline seizure frequency (Seizure/month)	Gender	Age	Epilepsy duration	Onset age	Etiology
GSM6725527	1	M	26	4	22	Unk
GSM6725534	60	M	19	7	12	TBI
GSM6725528	0.33	F	32	8	24	CVA
GSM6725532	4	F	45	8	37	Abor
GSM6725529	4	M	16	10	6	Unk
GSM6725530	1	F	35	13	22	Unk
GSM6725524	3	M	38	17	21	Unk
GSM6725537	1	M	26	19	7	Unk
GSM6725536	2	M	32	25	7	Unk
GSM6725525	0.25	M	37	35	2	Unk
GSM6725531	1	F	54	36	18	Ecl
GSM6725538	4	M	45	37	8	Unk
GSM6725539	2	F	58	37	21	Unk
GSM6725533	2	M	46	43	3	Unk
GSM6725526	0.25	M	60	47	13	Unk
GSM6725535	2	F	62	61	1	Inf

Clinical information in this table was adapted from original publications [16, 17]. *TBI* traumatic brain injury, *Unk* unknown, *CVA* stroke, *Abor* abortion, *Inf* infection, *Ecl* preeclampsia.

**Table 2:** Descriptive statistics of epilepsy duration

Epilepsy duration		Long (> 20 yr)	Short (≤ 20 yr)
	**N**	8	8
**Duration**	**mean**	40.12	10.75
	**std dev**	10.58	5.18
	**min**	25	4
	**max**	61	19
**Baseline seizure frequency**	**mean**	1.69	9.29
	**std dev**	1.22	20.54
	**min**	0.25	0.33
	**max**	4	60
	***P*** **value**	0.45	
**Age**	**mean**	49.25	29.63
	**std dev**	11.04	9.75
	**min**	32	16
	**max**	62	45
	***P*** **value**	0.0073	
**Onset age**	**mean**	9.13	18.88
	**std dev**	7.51	10.23
	**min**	1	6
	**max**	21	37
	***P*** **value**	0.0515	
**Gender**	**F**	3	3
	**M**	5	5
**Seizure group (%)**	**high**	12.5	50
	**low**	87.5	50

Patients were stratified into short-duration (≤ 20 years) and long-duration (> 20 years). The two groups were statistically compared for epilepsy duration, baseline seizure frequency (seizure/month), age, onset age, gender. Low seizure group represent seizure frequency ≤ 2 seizures/month and high seizure group represent seizure frequency > 2 seizures/month.

Further, correlation analysis identified significant positive correlation of 9 of the 18 upregulated genes with epilepsy duration (Fig. 1D) after excluding age-associated genes. In contrast, 2 of 10 downregulated genes were significantly inversely correlated with epilepsy duration (Fig. 1E), reinforcing their relevance to disease progression. These findings suggest that prolonged epilepsy is associated with peripheral transcriptional changes indicative of elevated oxidative stress and impaired RNA/DNA maintenance mechanisms. Such changes may reflect systemic effects of chronic seizure activity, long-term treatment exposure, or other disease-associated factors, although the underlying causes and clinical significance require further investigation.

### Correlation Analysis Identified Genes Associated with Epilepsy Duration

**Fig. 2:**
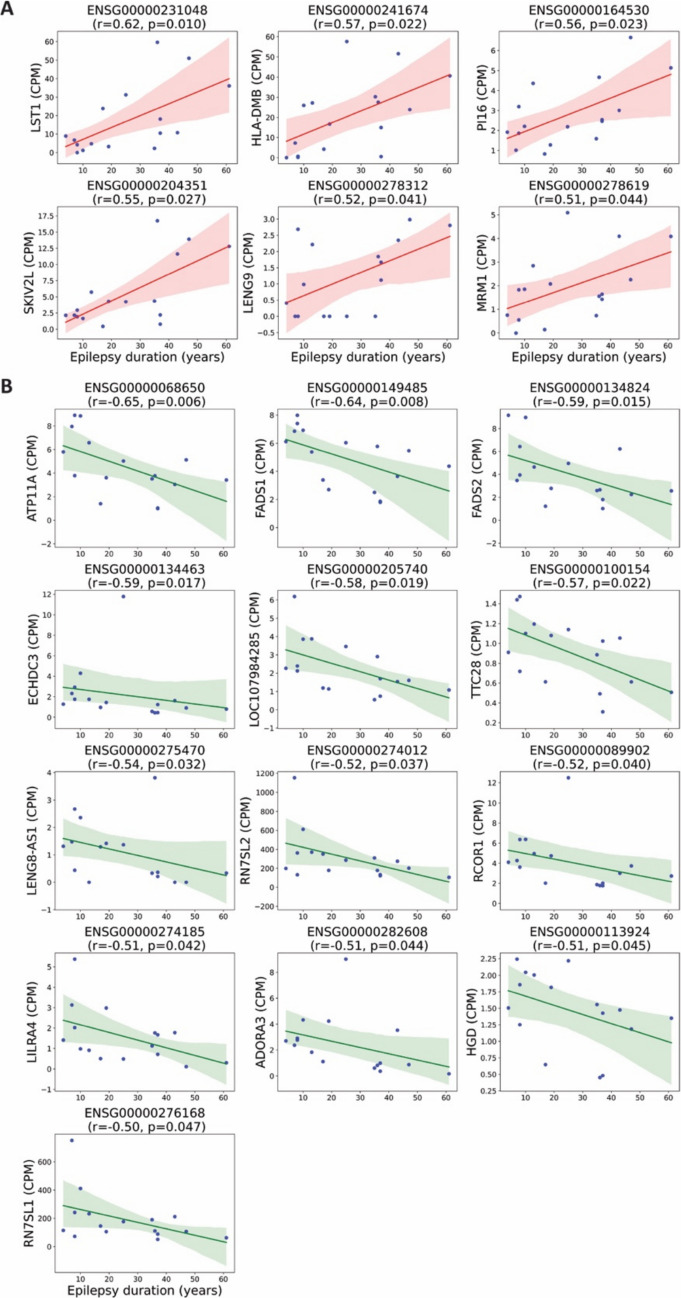
Spearman correlation between gene expression and epilepsy duration. Spearman correlation analysis was performed between epilepsy duration and the expression levels of all 11,929 expressed genes. This figure highlights a subset of significant genes not included in the differential expression analysis shown in Fig. 1. Only non-age-associated genes were visualized. Each plot shows the regression line with a shaded area indicating the 95% confidence interval. r: Spearman correlation coefficient. (**A**) Genes positively correlated with epilepsy duration. (**B**) Genes negatively correlated with epilepsy duration

To further characterize the transcriptomic relationship with epilepsy chronicity, we performed a Spearman correlation analysis between epilepsy duration and the expression levels of all 11,929 expressed genes. This analysis identified 15 genes with significant positive correlation with disease duration, six of which were not differentially expressed in the group-based comparison (Fig. 2A, Suppl. data file C). Additionally, 19 genes were significantly negatively correlated with duration, of which 16 were uniquely identified by correlation analysis and not captured by the differential expression approach. Three genes were excluded as they overlapped with age-associated genes. (Fig. 2B, Suppl. data file D). This unbiased approach identified additional 13 genes whose expression levels were continuously associated with disease duration, beyond binary group comparisons. These findings highlight a continuous transcriptomic shift associated with longer epilepsy duration, suggesting progressive biological alterations in peripheral blood cells that may mirror central nervous system changes over time.

**Fig. 3:**
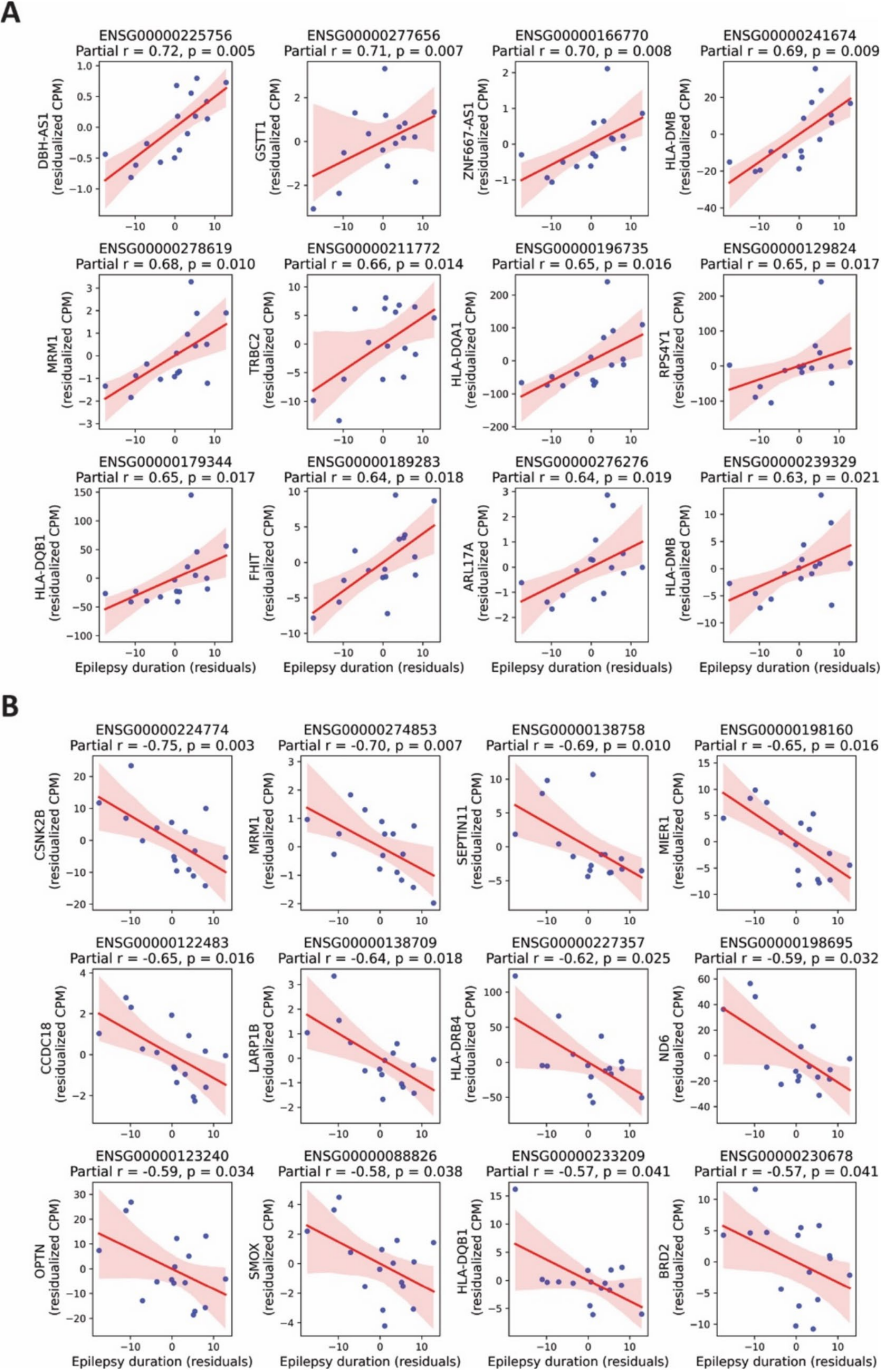
Partial Spearman correlation between gene expression and epilepsy duration. Partial Spearman correlation analysis was performed between gene expression and epilepsy duration, adjusting for age, gender, and etiology. This figure highlights top12 representative genes with highest partial Spearman correlation coefficient. Scatter plots show residualized gene expression versus residualized epilepsy duration. (**A**) Genes with positive partial correlation with epilepsy duration. (**B**) Genes with negative partial correlation with epilepsy duration

To account for potential confounding variables, we performed a partial Spearman correlation analysis between gene expression and epilepsy duration, adjusting for age, gender, and etiology. This analysis revealed 50 genes with a significant positive partial correlation with disease duration and 22 genes showing a significant negative partial correlation (Fig. 3A, B, Suppl. data file E and F). Notably, several of these genes were not identified in the unadjusted correlation analysis and differential expression analysis, suggesting that controlling for clinical covariates uncovered additional transcriptomic associations with epilepsy chronicity. These findings highlight a refined set of genes whose expression is independently associated with disease duration, reinforcing the presence of biologically relevant transcriptional alterations linked to the chronic course of epilepsy.

### Leukocyte Transcriptomic Landscape Differs Between ChOE and AOE

**Fig. 4:**
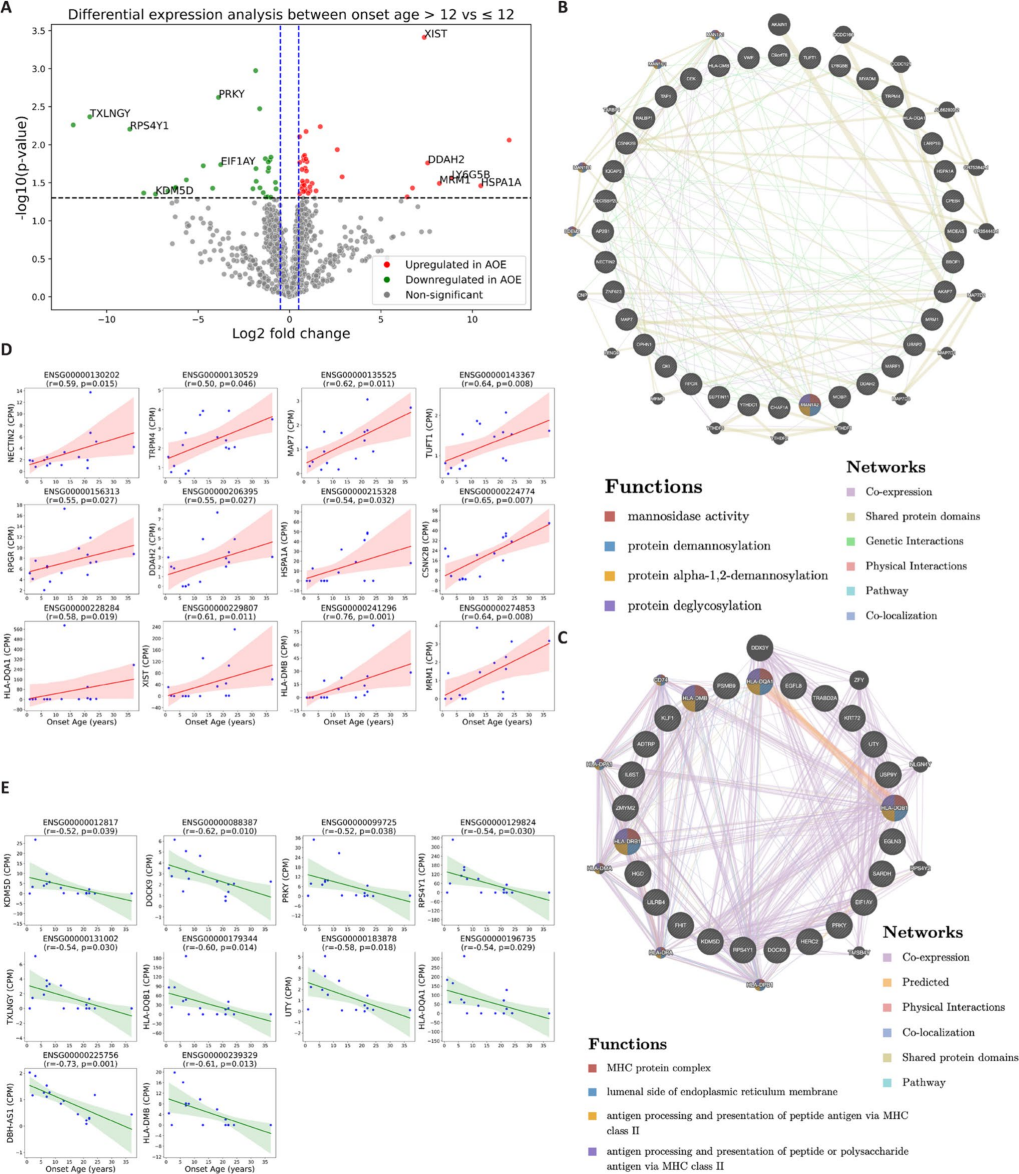
Leukocyte transcriptomic alterations associated with age at epilepsy onset. (**A**) Volcano plot showing differentially expressed genes in whole-blood leukocytes between patients with ChOE and AOE. Significantly upregulated genes in the AOE (log₂ fold change > 0.5, *p*-value < 0.05) are highlighted in red, while significantly downregulated genes are shown in green. Top 5 upregulated and downregulated non-HLA genes are annotated. (**B-C**) Functional association network analysis of significantly upregulated (**B**) and downregulated (**C**) genes using GeneMANIA (https://genemania.org/). Input (query) genes are indicated by striped inner nodes. The top four enriched functional terms (based on FDR) are displayed. (**D-E**) Spearman correlations between expression levels of upregulated (**D**) and downregulated genes (**E**) and onset-age. Only non-age-associated genes were visualized. Red and green lines indicate linear regression, and shaded areas represent 95% confidence intervals. *r*: Spearman correlation coefficient

To investigate how age at epilepsy onset influences the peripheral transcriptome, patients were stratified into two groups based on age of seizure onset: ChOE: ≤ 12 years and AOE: > 12 years with 8 patients in each group. The mean onset-age was 5.75 years in the ChOE group and 22.25 years in the AOE group. The age, baseline seizure frequency and epilepsy duration were comparable between the groups (Table 1, 3), minimizing potential confounders. Differential gene expression analysis of whole-blood leukocyte RNA-seq data identified 37 genes significantly upregulated (log₂ fold change > 0.5) and 31 genes significantly downregulated in the AOE group compared to the ChOE (Fig. 4A, Suppl. data file G and H). Notably, 11 of the significantly differentially regulated genes associated with age at epilepsy onset—CSNK2B, TAP1, DDAH2, EGFL8, HLA-DMB, HLA-DQA1, HLA-DQB1, HLA-DRB1, LY6G5B, PSMB9 and HSPA1A—are located within the EJM1 locus on chromosome 6p21 (supp. Figure 1B). Functional association network analysis of upregulated genes revealed overrepresentation of pathways related to mannosidase activity and protein deglycosylation. In contrast, downregulated genes showed enrichment of MHC protein complex, antigen processing and presentation (Fig. 4B, C), suggesting differential immune-related transcriptional activity between childhood and adolescent onset cases. Notably, after excluding age-associated genes, 12 of the 37 upregulated genes also showed significant positive correlation with onset-age (Fig. 4D), while 10 of 31 downregulated genes were significantly inversely correlated with onset-age (Fig. 4E), reinforcing their potential relevance to early disease manifestation.

**Table 3:** Descriptive statistics of onset-age

Onset age		ChOE (≤ 12 yr)	AOE (> 12 yr)
	**N**	8	8
**Onset age**	**mean**	5.75	22.25
	**std**	3.62	6.84
	**min**	1	13
	**max**	12	37
**Baseline seizure frequency**	**mean**	9.41	1.57
	**std dev**	20.48	1.33
	**min**	0.25	0.25
	**max**	60	4
	**P value**	0.24	
**Age**	**mean**	35.38	43.50
	**std dev**	15.40	12.74
	**min**	16	26
	**max**	62	60
	**P value**	0.34	
**Duration**	**mean**	29.63	21.25
	**std dev**	18.04	16.32
	**min**	7	4
	**max**	61	47
	**P value**	0.40	
**Gender**	**F**	1	5
	**M**	7	3
**Seizure group (%)**	**high**	37.5	25
	**low**	62.5	75

Patients were stratified into childhood onset epilepsy (ChOE, ≤ 12 year) and adolescent onset epilepsy (AOE, > 12 year). The two groups were statistically compared for epilepsy duration, baseline seizure frequency (seizure/month), age, onset age, gender. Seizure frequency was categorized as low (≤ 2 seizures/month) or high (> 2 seizures/month).

### Correlation Analyses Reveal Continuous Transcriptomic Associations with Seizure Onset-Age

**Fig. 5:**
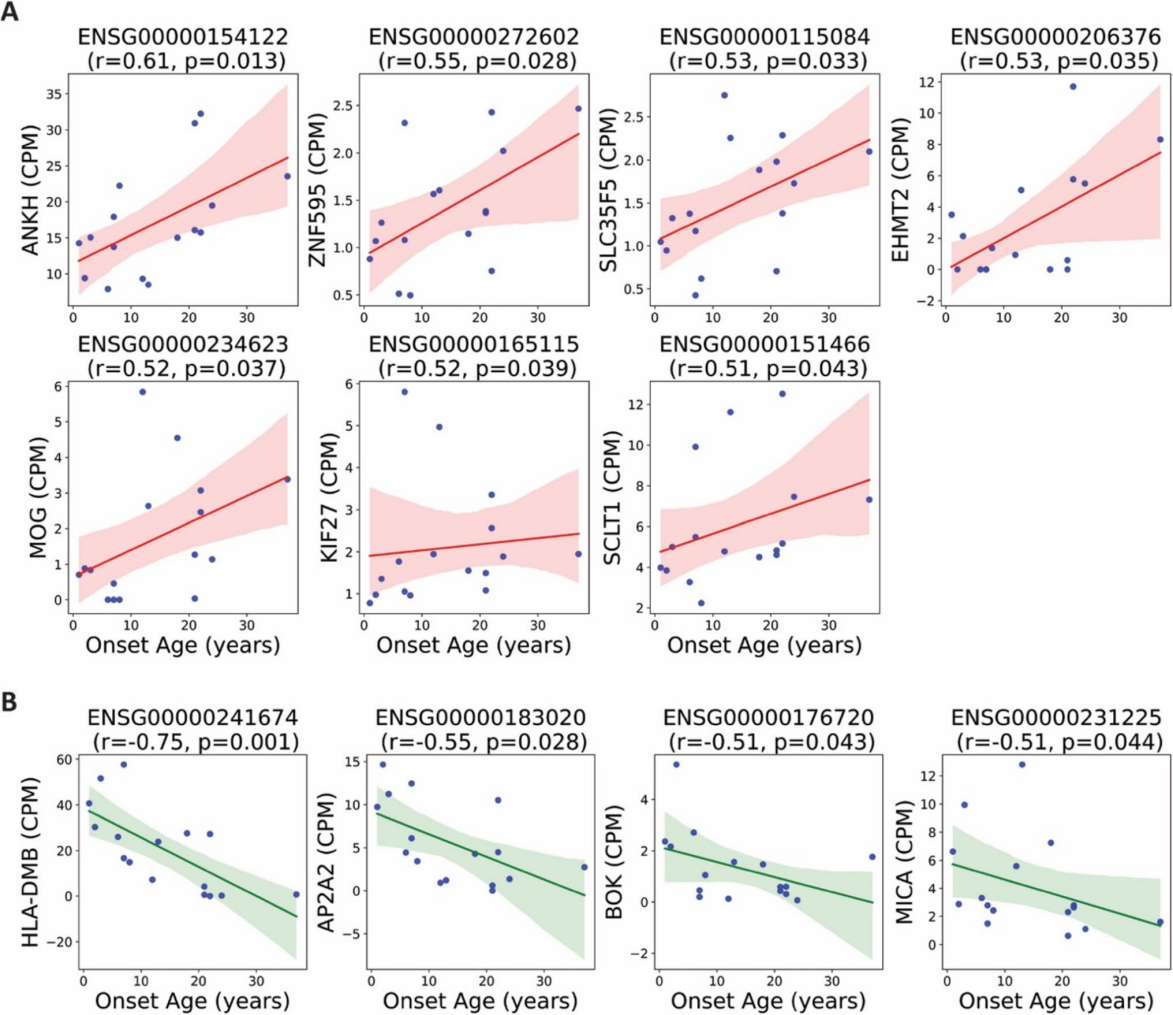
Spearman correlation between gene expression and age of epilepsy onset. Spearman correlation analysis was performed between age of epilepsy onset and the expression levels of all 11,929 expressed genes. This figure highlights a subset of significant genes not included in the differential expression analysis shown in Fig. 4. Only non-age-associated genes were visualized. Each plot shows the regression line with a shaded area indicating the 95% confidence interval. r: Spearman correlation coefficient. (**A**) Genes positively correlated with onset-age. (**B**) Genes negatively correlated with onset-age

To further characterize the transcriptomic relationship with onset-age, we performed a Spearman correlation analysis between onset-age and the expression levels of all 11,929 expressed genes. This analysis identified 22 genes with significant positive correlation with onset-age, seven of which were non-age-associated and not differentially expressed in the group-based comparison (Fig. 5A, Suppl. data file I). Additionally, 14 genes were significantly negatively correlated with onset-age, of which four were uniquely identified by correlation analysis and not captured by the differential expression approach (Fig. 5B, Suppl. data file J). These findings may suggest that some transcriptomic changes evolve gradually with age of onset.

**Fig. 6:**
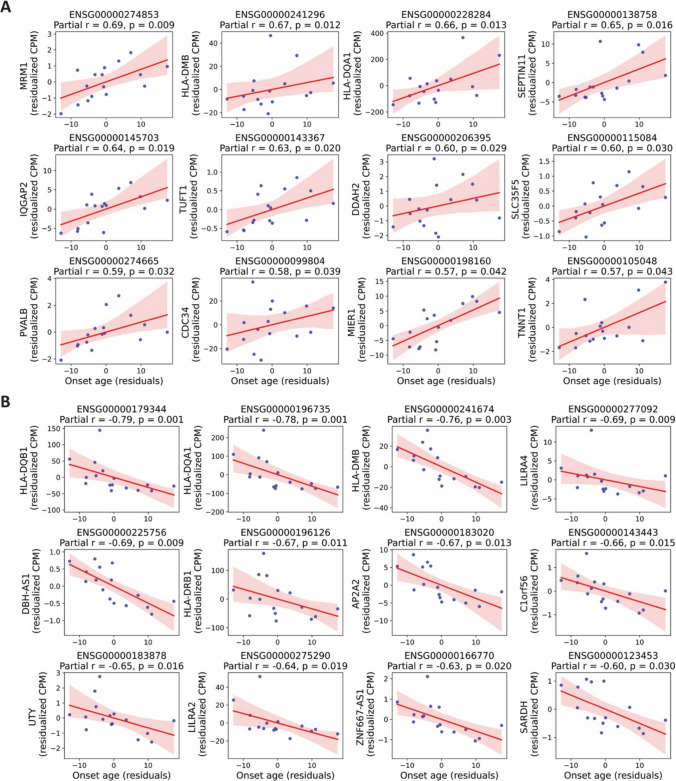
Partial Spearman correlation between gene expression and age of epilepsy onset. Partial Spearman correlation analysis was performed between gene expression and age of epilepsy onset, adjusting for age, gender, and etiology. This figure highlights top12 representative genes with highest partial Spearman correlation coefficient. Scatter plots show residualized gene expression versus residualized onset age. (**A**) Genes with positive partial correlation with age of epilepsy onset. (**B**) Genes with negative partial correlation with age of epilepsy onset

To control potential confounding variables, we performed a partial Spearman correlation analysis between gene expression and onset-age, adjusting for age, gender, and etiology. This analysis revealed 17 genes with a significant positive partial correlation with onset-age and 21 genes showing a significant negative partial correlation (Fig. 6A, B, Suppl. data file K and L). These results further support the existence of distinct and progressive transcriptomic signatures related to the timing of epilepsy onset.

Together, these findings indicate that the peripheral immune and transcriptional landscape differs between childhood- and adolescent-onset epilepsy and vary with age at seizure onset. The observed enrichment of immune-related processes suggests a potential long-term imprint of early neuroimmune interactions on systemic gene expression patterns; however, the directionality, causality, and long-term implications of this relationship remain unclear.

## Discussion

In this study we analyzed molecular correlates of epilepsy duration and onset age in a leukocyte transcriptome. Epilepsy and its treatment can exert systemic effects beyond the brain, it is important to study the effect of epilepsy on physiological systems to understand clinical trajectory of disease. Epilepsy duration is an interesting parameter, reflecting exposure to disease as well as treatment. This study of transcriptome analysis of leukocytes with short vs long epilepsy duration and ChOE vs AOE identified many differentially expressed, epilepsy duration- and onset age- correlated genes in leukocytes. Three genes (MRM1, HLA-DMB, TAP1) were differentially expressed in both the age at onset and epilepsy duration analyses. Given the inverse relationship between age at onset and epilepsy duration- where earlier onset generally corresponds to longer disease duration- it is notable that these genes were upregulated in the age of onset analysis but downregulated in individuals with longer epilepsy duration. Consistent with this opposing relationship, genes positively correlated with epilepsy duration were compared with those negatively correlated with age at onset, identifying 17 overlapping genes (BOK, HLA-DMB, MRM1, DOCK9, RPS4Y1, SLC14A1, ZNF667-AS1, HLA-DQB1, H2AW, COL18A1, UTY, FHIT, HLA-DRB1, HLA-DQA1, DBH-AS1, ARL17A, LILRA4). Conversely, comparing genes negatively correlated with epilepsy duration with those positively correlated with age at onset revealed nine overlapping genes (TAP1, MRM1, SLC35F5, MAP7, LARP1B, SEPTIN11, MIER1, CSNK2B, MOG). These findings may indicate partially overlapping but directionally distinct molecular processes associated with disease onset and chronicity. We also observed a small subset of seizure outcome-related genes (CPEB4, AKAP7, FADS2) from the prior analysis of this dataset [17] overlapping with our analysis, suggesting potential roles in epilepsy onset and progression.

Several genes identified in our analysis reflect dynamic neuro-immune molecular changes linked to epilepsy onset age and disease duration. The CSNK2B, a regulator of early brain development, is mutated in Poirier-Bienvenu neurodevelopmental syndrome, characterized by childhood onset epilepsy, developmental delay, and behavioral issues (meta-analyzed in [23]). Intriguingly, we observed increased CSNK2B expression in individuals with AOE, which may reflect a compensatory or adaptive response in chronic disease. OPHN1, which involved in synaptic development as well as platelet cytoskeletal reorganization and activation, is well established as a cause of X-linked intellectual disability and epilepsy [24, 25]. In our study, OPHN1 was upregulated in individuals with AOE, suggesting a possible link between neuronal homeostasis and peripheral immune-vascular responses. Similarly, TRPM4 was upregulated in peripheral leukocytes of individuals with AOE. TRPM4 is a calcium-activated cation channel expressed in hippocampal mossy cells, where it regulates neuronal excitability and contributes to seizure susceptibility [26]. We also observed increased expression of HSP70 (HSPA1A), a stress-response protein with context-dependent roles in epilepsy. Elevated CSF levels of HSP70 have been associated with status epilepticus [27], and mouse studies suggest that HSP70 can both promote seizure initiation and protect against excessive neuroinflammation [28]. Its leukocyte upregulation in AOE may suggest a potential link between systemic stress responses and seizure burden. NFIX, a neurodevelopmental gene previously associated with epilepsy, was upregulated in individuals with longer disease duration, suggesting broader involvement in transcriptional regulation during chronic epilepsy [29, 30].

In our study, we observed that GABBR1 was significantly upregulated in individuals with longer epilepsy duration, aligning with its established role in inhibitory neurotransmission and seizure susceptibility [31–33]. Beyond its neural function, GABBR1 is also expressed in hematopoietic stem and progenitor cells (HSPCs), where it regulates cell proliferation and homeostasis [34], thus pointing to potential neuroimmune crosstalk in epilepsy pathophysiology. GABBR1 resides within the EJM1 locus on chromosome 6p21, a well-established susceptibility region for JME [22]. Several additional genes located within this locus were also found to be differentially expressed in our analysis. These genes may act synergistically to modulate neuroimmune dynamics. For example, prior studies have identified associations between TAP1 polymorphisms or haplotypes and increased risk of JME or idiopathic generalized epilepsy across diverse populations [35, 36]. Several recent studies point to the complexity of polygenic influence in epilepsy [37, 38]. Our finding that genes associated with genetic generalized epilepsy are enriched in childhood-onset epilepsies fits well with such models. Taken together, these findings support a model in which the EJM1 locus contributes to epilepsy risk and progression through both neuronal and immune-mediated mechanisms.

Emerging evidence implicates dynamic post-transcriptional regulation as a critical mechanism in epilepsy. CPEB4, a regulator of cytoplasmic polyadenylation, was upregulated in peripheral leukocytes of individuals with AOE. CPEB4 has been shown to modulate mRNA poly(A) tail length during epileptic activity [39, 40]. Elevated CPEB4 expression in both seizure models and resected hippocampal tissue from patients with drug-resistant TLE, along with our findings of increased expression in AOE, point to a potential adaptive role for CPEB4 in modulating disease progression over time. In contrast, CNOT3, a subunit of the CCR4-NOT complex involved in mRNA degradation and translational repression, was downregulated in individuals with longer epilepsy duration. Mutations in CNOT3 have been associated with intellectual disability, autism, and developmental epileptic encephalopathy [41], supporting its role in neuronal gene regulation. Together, these findings underscore the importance of RNA-binding proteins and mRNA metabolism in epilepsy.

Among genes upregulated in longer epilepsy duration, KCNN4 is notable for its roles in both neuronal and immune function. KCNN4 encodes a calcium-activated potassium channel KCa3.1, that regulates neuronal excitability and membrane potential. Its expression in excitatory neurons has been explored in gene therapy models to reduce seizure activity, pointing to its potential anticonvulsant properties [42, 43]. These findings are further supported by studies proposing KCNN4 as a therapeutic target in epilepsy [44, 45]. Beyond brain, KCNN4 influences T-cell activation and cytokine signaling, and its inhibition promotes anti-inflammatory responses [46, 47]. The convergence of neuronal and immune functions suggests KCNN4 may link neuroinflammation and epileptogenesis, particularly in chronic epilepsy.

Together, these findings underscore a broader model in which genes involved in synaptic regulation (OPHN1, TRPM4, GABBR1), stress response (HSPA1A), developmental signaling (CSNK2B, NFIX), and immune modulation (TAP1, HLA-DMB, LST1) interact to shape the course of epilepsy. Gene expression changes in peripheral immune cells- particularly in individuals with adolescent-onset or long-duration epilepsy- may reflect chronic adaptations, compensatory responses, or ongoing neuroimmune crosstalk, offering novel insights into systemic biomarkers and potential therapeutic targets.

## Limitations

Limitation of this study include relatively small sample size that may limit statistical power and the generalizability of findings. As a result, the analyses should be viewed as exploratory and hypothesis-generating, and larger cohorts (e.g., ≥ 50 individuals per category) will be required to robustly detect subtle effects. Second, leukocytes represent a highly dynamic and heterogeneous cell population influenced by circadian timing, transient inflammatory states (e.g., subclinical infections or allergies), comorbidities, and pharmacological exposures, including long-term anti-seizure medication therapy. Although we adjusted for key demographic and clinical variables available in this cohort, detailed information on blood draw timing, transient immune states, ASMs, dosages, and treatment duration was not available and therefore could not be explicitly modeled. Consequently, some of the observed transcriptomic changes may reflect chronic medication-related immune modulation rather than disease effects alone, and leukocyte gene expression may provide indirect insights into long-standing treatment exposure.

Third, bulk RNA-seq was performed on blood leukocytes, which may dilute cell-type-specific signals and obscure subtle immune alterations. In addition, the results are correlative and require replication in independent cohorts and validation using orthogonal methods such as single-cell transcriptomics or proteomics. Additionally, in patients with ChOE, the immune-related transcriptomic changes may be driven by underlying genetic variants affecting the immune system, suggesting that these changes could be a cause rather than a consequence of epilepsy. Longitudinal sampling and integration with germline genetic data could help disentangle causality. Finally, as peripheral blood may not fully mirror central nervous system processes, functional studies are needed to clarify the biological relevance of these systemic molecular signatures.

## Conclusion

Our findings identify distinct leukocyte transcriptomic differences associated with both age of onset and epilepsy duration, including changes in immune-related and oxidative stress pathways. The enrichment of differentially expressed genes within the EJM1 locus may further suggests a coordinated genetic influence on epilepsy development. These results highlight that peripheral blood profiling could help explore biomarkers and biological processes related to disease onset and progression.

## Supplementary Information

Below is the link to the electronic supplementary material.

Supplementary file1 (XLSX 32 KB)

Supplementary file2 (DOCX 1259 KB)

## Data Availability

No datasets were generated or analysed during the current study.
