# Supplementary material for: Leukocytes Transcriptome Analysis of Genes Associated with Epilepsy Duration and Age of Onset
Source: Mol Neurobiol. 2026 Apr 8;63(1):550. doi: 10.1007/s12035-026-05845-5 (PMC13061771; doi:10.1007/s12035-026-05845-5)
Supplement: Supplementary file 2 — Supplementary file2 (DOCX 1259 KB) [file 12035_2026_5845_MOESM2_ESM.docx]

**Leukocytes transcriptome analysis of genes associated with epilepsy duration and age of onset**

**
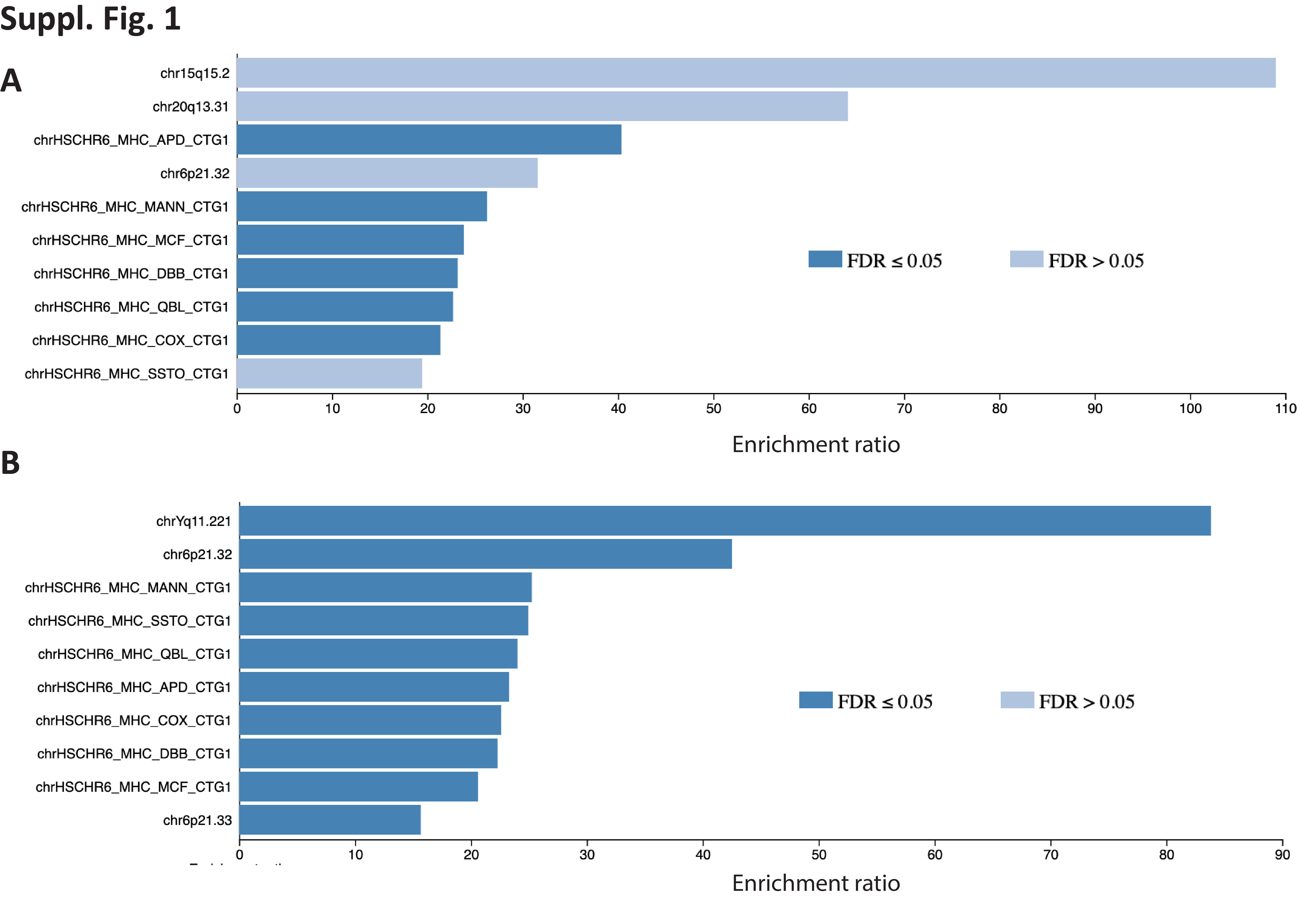
**

**Suppl. Figure 1: Chromosomal location and cytogenetic band of differential level genes associated with long epilepsy duration and onset age. (A)** List of up and downregulated genes in long epilepsy duration were analyzed using WebGestalt (<https://www.webgestalt.org/>) [21] with genome as a reference set. Cytogenetic bands enriched for these genes are displayed, with statistically significant regions (FDR < 0.05) highlighted in dark blue. **(B)** Up- and downregulated genes associated with age at epilepsy onset were analyzed using WebGestalt using the same parameters as in panel A.
